# Gliding locomotion of manta rays, killer whales and swordfish near the water surface

**DOI:** 10.1038/s41598-017-00399-y

**Published:** 2017-03-24

**Authors:** Jie-Min Zhan, Ye-Jun Gong, Tian-Zeng Li

**Affiliations:** Department of Applied Mechanics and Engineering, School of Engineering, Sun Yat-sen University, Guangzhou, 50275 China

## Abstract

The hydrodynamic performance of the locomotive near the water surface is impacted by its geometrical shape. For marine animals, their geometrical shape is naturally selective; thus, investigating gliding locomotion of marine animal under the water surface may be able to elucidate the influence of the geometrical shape. We investigate three marine animals with specific geometries: the killer whale is fusiform shaped; the manta ray is flat and broad-winged; and the swordfish is best streamlined. The numerical results are validated by the measured drag coefficients of the manta ray model in a towing tank. The friction drag of the three target models are very similar; the body shape affected form drag coefficient is order as swordfish < killer whale < manta ray; the induced wave breaking upon the body of the manta ray performs different to killer whale and swordfish. These bio-inspired observations provide a new and in-depth understanding of the shape effects on the hydrodynamic performances near the free surface.

## Introduction

Hydrodynamic drag consists of three primary components: form drag, friction drag and wave drag^1^. Form drag arises from pressure differences over the moving body and depends primarily on the body shape. Friction drag is caused by fluid viscous shear over the body surface. When the form drag is considerably greater than the friction drag, the body is termed blunt; otherwise, the body is termed streamlined. Wave drag occurs when the moving body is near the water surface, pushing the water out of its way and creating waves. The wave drag reflects the primary effect of the free surface on the hydrodynamic performance of the moving body.

When autonomous underwater vehicle (AUV) glides under the water surface (also known as the free surface), the wavy deformation of the free surface will affect the vehicle operation: the induced wave drag adds up to increase the hydrodynamic resistance^2^, and the free surface may enhance the vehicle's directional stability^3^. The effect of the free surface is significant for underwater vehicles that are operated in shallow water. However, the hydrodynamic performance of vehicles near the free surface is affected by numerous factors, including wind-generated waves, thruster motion, and cavitation bubbles from high-speed relative motion. The free surface effect cannot be easily defined because it can take various forms, as noted above. This study focuses on the wave-associated free surface effect and its relationship with the geometrical shape of the vehicle. It is expected to provide insight into the shape selection of underwater vehicles for littoral applications.

To adapt to the living environment, the external shape of marine animal has changed slowly by natural evolution. Some of the evolved geometric characteristics may inspire the underwater vehicle optimal design. For example, the streamlined shape of the whale inspired the design of high-speed submarines in World War II^4^. Another example is the supersonic aircraft whose needle-like nose, similar to that of the swordfish, is designed to reduce the shock drag at the supersonic speed. A recent example is the manta ray which is diamond-shaped and well known for its strong gliding ability. A biomimetic manta ray robot can perform steady gliding and can pivot turning and backward locomotion, making it suitable for carrying high-precision measurement equipment^5^. The biorobotic autonomous undersea vehicles, especially the robotic fish, have attracted wide research attention^6–8^. As mentioned above, the wave drag is one important factor affecting the hydrodynamic performance of underwater vehicle. However, few research has concerned about the hydrodynamic drag of a marine animal near the free surface.

In laboratory, it is hard or even impossible to measure the drag force of a living marine animal under fixed postures without any damage. Here, we build a physical model of the manta ray using the 3D printing technology, such that it is able to provide non-invasive measurement data of the 3D manta ray model. Additionally, experimental measurements are costly expensive and unable to provide full-field data. Computational fluid dynamics (CFD) provide a tool to simulate the flow around the body in computer, and the CFD technology develops quickly with the development of the parallel computation and super-computer technology. Mittal^9^ reviewed numerous applications of CFD techniques in bio-hydrodynamics^9^. Limited by computation condition, most previous CFD studies have focused on local details, e.g., various descriptions have been used to model the fin flapper locomotion for a small compartment from a 2D rigid airfoil^10^ to a 3D flexible wing^11^. Park *et al.* (2014) simulated the full-body hydrodynamics of a jellyfish with paddling-based locomotion using a simplified 3D bell shape geometry which is discretized into 6144 triangular elements, and the whole computation domain is based on a 128 × 256 × 128 uniform mesh. The numerical simulation of the flow around a large size marine animal requires considerably higher computation cost.

The computational complexity is also increased by accounting for the free surface effect. An additional continuity equation for the liquid phase volume fraction has been introduced to solve the air-water interface: this volume of fraction (VOF) method is one of the most popular free surface tracking methods^12^. Jagadeesh *et al.* simulated a regular body near the water surface using a Reynolds-averaged Navier-Stokes (RANS) solver coupled with the VOF method and obtained results those were in good agreement with the experimental data^13^. Recently, Zhan *et al.* used the same approach in CFD simulations for the flow around a swimmer beneath the water surface, and the calculated drag forces on the swimmer were also consistent with the experimental results^14^. Here, the same measuring technique is used to obtain the hydrodynamic drag force of the 3D model at the SYSU (Sun Yat-sen University) Tow Tank.

**Figure 1:**
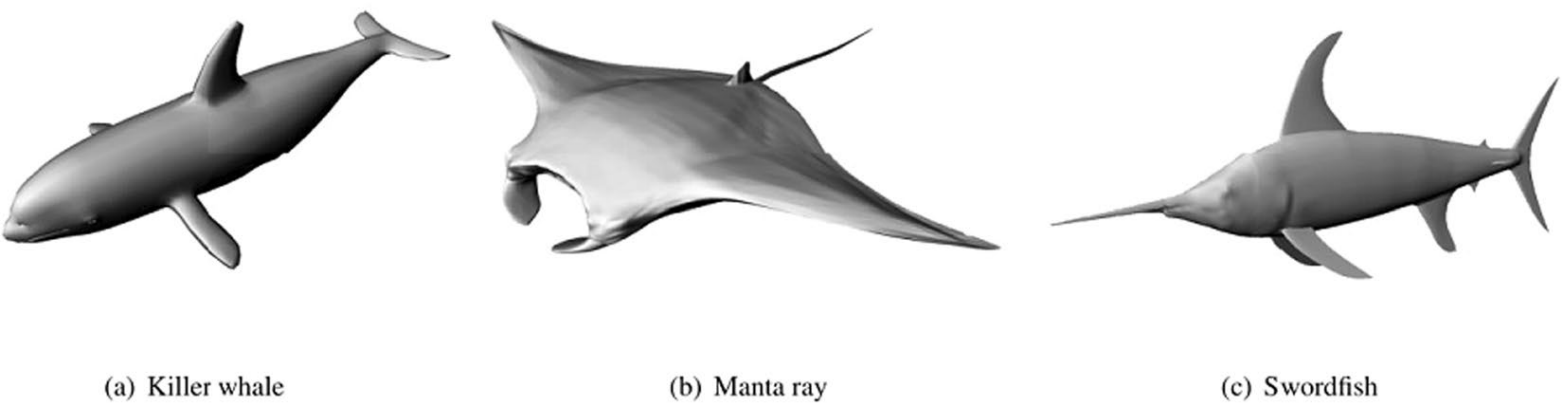
CAD models of the three marine animals, created in Rhinoceros^17^.

In this study, we focus on the marine animal gliding locomotives near the water surface, especially the influence of geometrical shape on its hydrodynamic performance. The following three types of marine animal are selected for their specific geometries, as shown in Fig. 1. A killer whale (Orcinus orca) has a large volume, and its streamlined fusiform shape is highly efficient for swimming^15^. The flattened diamond-shaped manta ray (Mobulidae: devil rays) is able to glide gracefully through the sea and has recently attracted considerable interest^16^. The manta ray is known as a ‘devilfish’ because of its ‘evil’ horn-shaped cephalic fins and ‘ghostly’ behaviors, such as being able to twist a boat around by pulling at the anchor. The third one, swordfish (Xiphi gladius), is also one of the well known high-speed marine animals, and its elongated body shape and needle-like nose are similar to several other high-speed marine animals including the sailfish (Perciformes) and the wahoo (Acanthocybium solandi).

The swimming speed of the three target objects are estimated and ordered as: swordfish (90 *km*/*h* ^18^) > killer whale (56 *km*/*h* ^19^) > manta ray (24 *km*/*h* ^20^). Note that the actual swimming speed of one marine animal is depend on the its age, size and species, so the above numbers are shown for reference only. Additionally, even if belongs to one species, the body size, shape and living characteristics still have wide variances. Here, we only concentrate on the obvious geometry characteristics of the three marine animals. For a better comparison, we build three target models with the same length (1 m), based on the reliable photographs and videos of the three marine animals on the internet.

Fluid flows around the three target models are numerically simulated in still water at various swimming speeds (0.5 *m*/*s*, 1 *m*/*s*, $\cdots$, 4 *m*/*s*) when the model is fully submerged (this scenario is known as Case UnderWater and is abbreviated as UW) or near the water surface (this scenario is known as Case NearSurface and is abbreviated as NS). To validate the numerical results, one 1 m length model of the manta ray is built using the 3D printing technology. The simulated drag force of the manta ray model is consistent with the experimentally measured drag force in a 6 m wide Towing Tank (Department of Applied Mechanics and Engineering, School of Engineering, Sun Yat-sen University)^14^. Friction drag, form drag and wave drag of the three target objects are calculated and compared. Swimming behavior under the water surface generates waves on the surface, and noticeable differences among the three targets are observed in the simulated wave profiles. The shape effects on the hydrodynamic resistances are analyzed and expected to provide biometric inspiration for the shape design of underwater vehicles used in littoral applications.

## Results

### Validation

**Figure 2:**
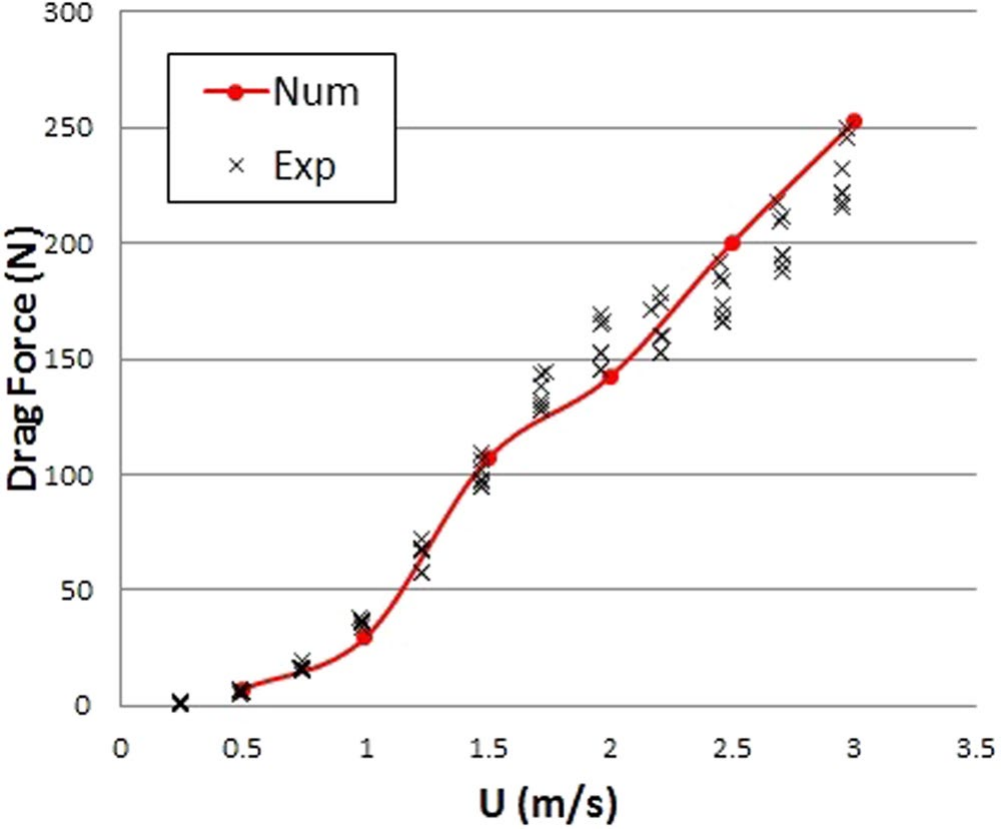
Comparison of the numerical simulated and experimental measured drag forces of the validation case.

To establish the credibility of the numerical method, we measured the drag force of the physical manta ray model in a towing tank at Sun Yat-sen University. Based on the CAD model used in the numerical simulation, the physical model is made of printed segments using one 3D printing machine (Ultimaker 2 extended+), and reinforced with the aluminum alloy frame. The manta ray model is hanging by four tear-shaped columns from the carriage, as explained in the Section of Experiment design. The position of the model is same to that in Case NearSurface (Table 1). Resistance tests of the manta ray model are repeated at least five times under each towing speed (0.25, 0.5, $\cdots$, 3.0 m/s). The relative numerical simulation is conducted at the same condition for the model with four tear-shaped columns. Note that the influence of the hanging columns on the drag force is not negligible. Hence the four tear-shaped columns are considered in the test model of the validation case in the numerical simulation. As shown in Fig. 2, the numerical simulated drag forces of the validation case are in good accordance with the experimental measurements.

**Table 1:** Test conditions for the three types of marine animals.

Name	Signs and Units	Killer whale	Manta ray	Swordfish
Wetted	*A* ^*UW*^[*m* ^2^]	0.8262	2.5323	0.6198
area	*A* ^*NS*^[*m* ^2^]	0.8153	2.5323	0.5985
Characteristic Length	*L*_0_[*m*]	1	1	1
Length	*L*[*m*]	1.165	2.124	1.605
Width	*W*[*m*]	0.546	2.232	0.165
Height	*H*[*m*]	0.38	0.279	0.613
Volume	*V*[*m* ^3^]	0.02693	0.13027	0.0123
Body	*d* ^*NS*^[*m*]	0.18	0.18	0.18
position	*d* ^*UW*^[*m*]	1.0	1.0	1.0
Water depth	*d*_*W*_[*m*]	2.5	2.5	2.5
Domain length	*L*_*D*_[*m*]	9	9	9
Domain width	*W*_*D*_[*m*]	6	6	6
Domain height	*H*_*D*_[*m*]	3.5	3.5	3.5
Mesh	*NS*[*Millions*]	4.205266	5.777767	3.883654
size	*UW*[*Millions*]	4.205266	6.805036	4.690324

### Hydrodynamic drag

Hydrodynamic drag force **F**_*D*_ is the sum of the pressure drag **F**_*p*_ and the friction drag **F**_*fr*_^1^. Pressure drag represents the magnitude of the pressure difference over the body in the flow direction. Size and shape of the body are the two principal factors that affect the drag force. To eliminate the size effect, the nondimensional drag coefficient is defined as *C*_*D*_ = 2**F**_*D*_/(*ρU* ^2^ *A*), where *U* is the swimming speed and *A* represents the wetted area. Then, the drag coefficient *C*_*D*_ can be written as the sum of the pressure drag coefficient *C*_*p*_ and the friction drag coefficient *C*_*fr*_:

1 $${C}_{D}={C}_{p}+{C}_{fr}=\frac{2{{\bf{F}}}_{p}}{\rho {U}^{2}A}+\frac{2{{\bf{F}}}_{fr}}{\rho {U}^{2}A}.$$

The value of the wetted area *A* is listed as *A* ^*UW*^ for Case UW and *A* ^*NS*^ for Case NS, as shown in Table 1). Furthermore, the pressure drag is assumed to be the sum of the form drag and wave drag. For Case UW, the wave drag is negligible, and then the pressure drag is assumed to equal to the form drag. Because the form drag depends primarily on the body shape, the form drag can be assumed to be constant whether in Case NS or Case UW. Hence, the wave drag coefficient can be approximated by:

2 $${C}_{w}\approx \frac{2({{\bf{F}}}_{p}^{NS}-{{\bf{F}}}_{p}^{UW})}{\rho {U}^{2}A}.$$

**Figure 3:**
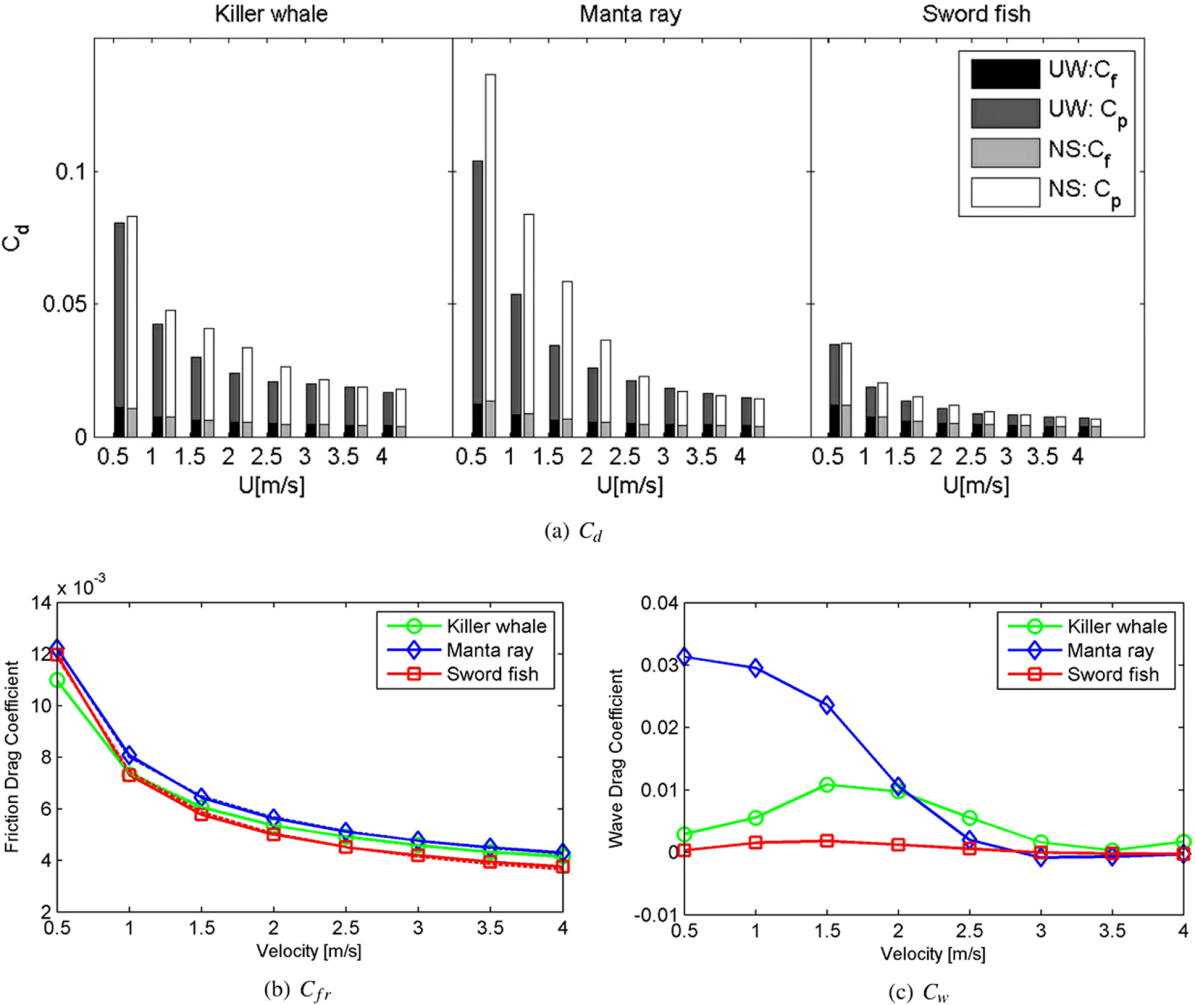
Drag coefficients at various swimming speeds: (**a**) left column: *C*_*D*_ for Case UW; right column: *C*_*D*_ for Case NS; top row: *C*_*p*_; bottom row: *C*_*fr*_; (**b**) *C*_*fr*_ for Case UW. Solid line: CFD simulation results; dashed line: fitting function in Eq. 3; (**c**) *C*_*w*_ for Case NS.

Calculated *C*_*D*_ values is shown in Fig. 3. Obviously, the value of drag coefficient *C*_*D*_ for the three models can be ordered as: manta ray > killer whale > swordfish. For killer whale and manta ray, the value of *C*_*D*_ in Case NS is greater than the relative value in Case UW, and the difference is mainly due to the wave drag, while this difference is not obvious for swordfish. Additionally, only for swordfish, the pressure drag is not the dominant contribution to the drag, which indicating that the swordfish is the best streamlined among the three marine animals.

#### Friction drag

Skin friction drag results from shear forces acting on the body surface, and as expected, the value of *C*_*fr*_ in Case NS is similar to that in Case UW for all three marine animals, as shown in Fig. 3(a). As shown in Fig. 3(b), the friction drag coefficient *C*_*fr*_ is nonlinearly dependent on the swimming speed. Furthermore, the relationship between *C*_*fr*_ and the Reynolds number had been noticed^21^. Here, the Reynolds number is defined as $Re=\frac{\rho U{L}_{0}}{\mu }$, where *ρ* is the fluid the density, *μ* the fluid viscosity and *L*_0_ = 1 *m* is the characteristic length of the model. Similar relationships between the friction drag coefficients and the Reynolds numbers are found for the three targets:

3 $${C}_{fr}^{UW}=\{\begin{array}{ll}0.0710R{e}^{-0.2}+2888.7150R{e}^{-1}, & {\rm{for}}\,{\rm{killer}}\,{\rm{whale}},\\ 0.0704R{e}^{-0.2}+3533.6204R{e}^{-1}, & {\rm{for}}\,{\rm{manta}}\,{\rm{ray}},\\ 0.0558R{e}^{-0.2}+3898.5099R{e}^{-1}, & {\rm{for}}\,{\rm{swordfish}}.\end{array}$$

The calculated *C*_*fr*_ values fit Eq. 3 very well, as shown in Fig. 3(b). The *Re* ^−0.2^ term fitting coefficient is sorted in the order: manta ray > killer whale > swordfish. The value of ${C}_{fr}^{UW}$ follows the same order when the swimming speed *U* ≥ 1 *m*/*s*. Overall, however, the differences of the friction drag coefficients between the three models are very small. Hence, it can be concluded that he different hydrodynamic performances of the three targets arise mainly from the differences in the pressure drag coefficient, as shown in Fig. 3(a).

#### Form drag

Form drag depends mainly on the geometrical shape; form drag in the deep sea is also known as pressure drag, i.e. ${C}_{fo}\cong {C}_{p}^{UW}$. As discussed above, the friction drag coefficients of the three models are very similar, and the form drag is the main factor causing the variation of the drag coefficients of the three target models. Figure 3(a) shows that the pressure drag coefficients of the three target models are the same order of drag coefficients: manta ray > killer whale > swordfish.

**Figure 4:**
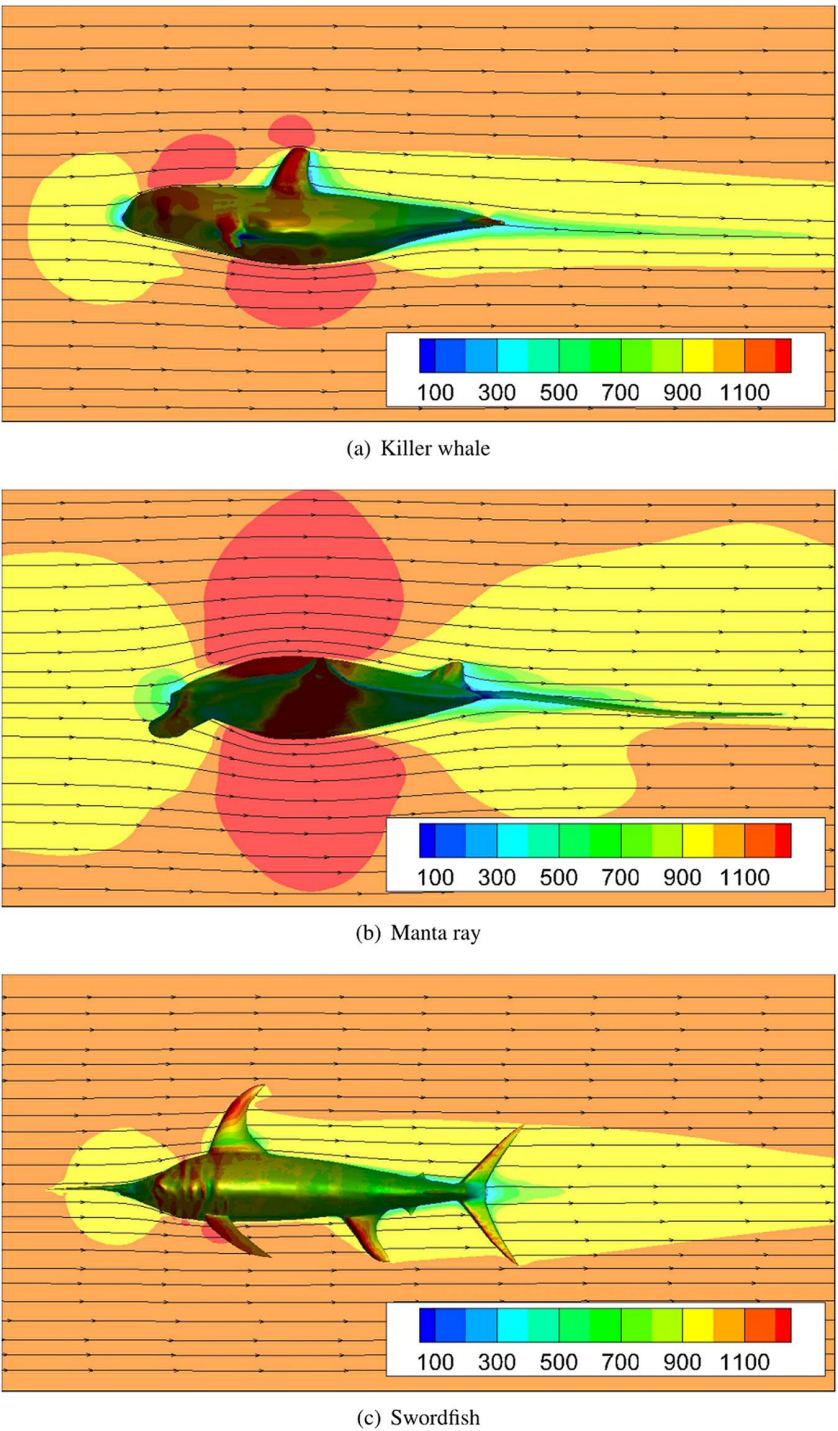
Dynamic pressure contour plots with streamlines at U = 4 m/s for Case UW. The CAD models are created in Rhinoceros^17^.

Marine animal models can be viewed as obstacles to fluid flow that creates boundary layer separation. Inside the turbulent boundary layer, the pressure is relatively low in the wake behind the obstacle. A non-streamlined shape has a wide wake, which generates attendant vorticity: a strong adverse pressure gradient results in a large pressure drag coefficient. Figure 4 shows the contour plot of the dynamic pressure along with the streamlines. Obviously, pressure differences around the bodies can be sorted in the same order as the pressure drag coefficients.

The geometrical shape of the model decides the value of its form drag. Sometimes, form drag represents the pressure drag, when the other components of the drag force are neglected. In Case UW, the scaled model is 1.0 m deep in water, and the wave drag can be neglected, such that the form drag is assumed to equal the pressure drag in Case UW. Because the friction drag coefficients of the swordfish is similar to killer whale and manta ray, it can be possible to conclude that the best streamlined body shape of the swordfish is the main reason that why it swims fastest among the three targets.

#### Wave drag

In Case NS, the model is near the free surface, and the wave induced by the body motion affect the pressure distribution over the body. Higher pressure drag coefficient $({C}_{p}^{NS} > {C}_{p}^{UW})$ are observed at certain swimming speeds, as shown in Fig. 3(a). The wave drag is negligible when the submergence depth of the underwater object is deep enough^13^. As mentioned before, the pressure drag is mainly composed by the form drag and the wave drag. The wave drag is near zero in deep water, i.e. ${C}_{w}^{UW}\approx 0$. And as mentioned above, the form drag in Case NS is similar to that in Case UW. It can be concluded that the difference of the drag coefficient between Case NS and Case UW is mainly due to the wave drag. Hence, the wave drag coefficient *C*_*w*_ can be approximated as the difference between ${C}_{p}^{UW}$ and ${C}_{p}^{NS}$, i.e.

4 $${C}_{p}^{NS}-{C}_{p}^{UW}\approx ({C}_{fr}^{NS}+{C}_{w}^{NS})-{C}_{fr}^{UW}\approx {C}_{w}^{NS}.$$

Figure 3(c) shows that *C*_*w*_ for killer whale and swordfish peaks at the critical swimming speeds, *U*_*c*_ = 1.5 *m*/*s*. In ship hydrodynamics, a peak in *C*_*D*_ is generally observed^22, 23^, and the peak velocity is typically considered to be an unfavorable number for initiation of wave breaking^24, 25^. And the value *C*_*w*_ of the killer whale is greater than that of the slimmer swordfish. For manta ray, *C*_*w*_ decreases with the swimming speed U. Note that it is possible that *C*_*w*_ of manta ray becomes smaller when *U* < 0.5 *m*/*s*. Additionally, *C*_*w*_ of manta ray is much greater than that of other two models when *U* < 2 *m*/*s*, but is similar or even smaller than that of swordfish when *U* ≥ 2.5 *m*/*s*.

### Free surface deformation

**Figure 5:**
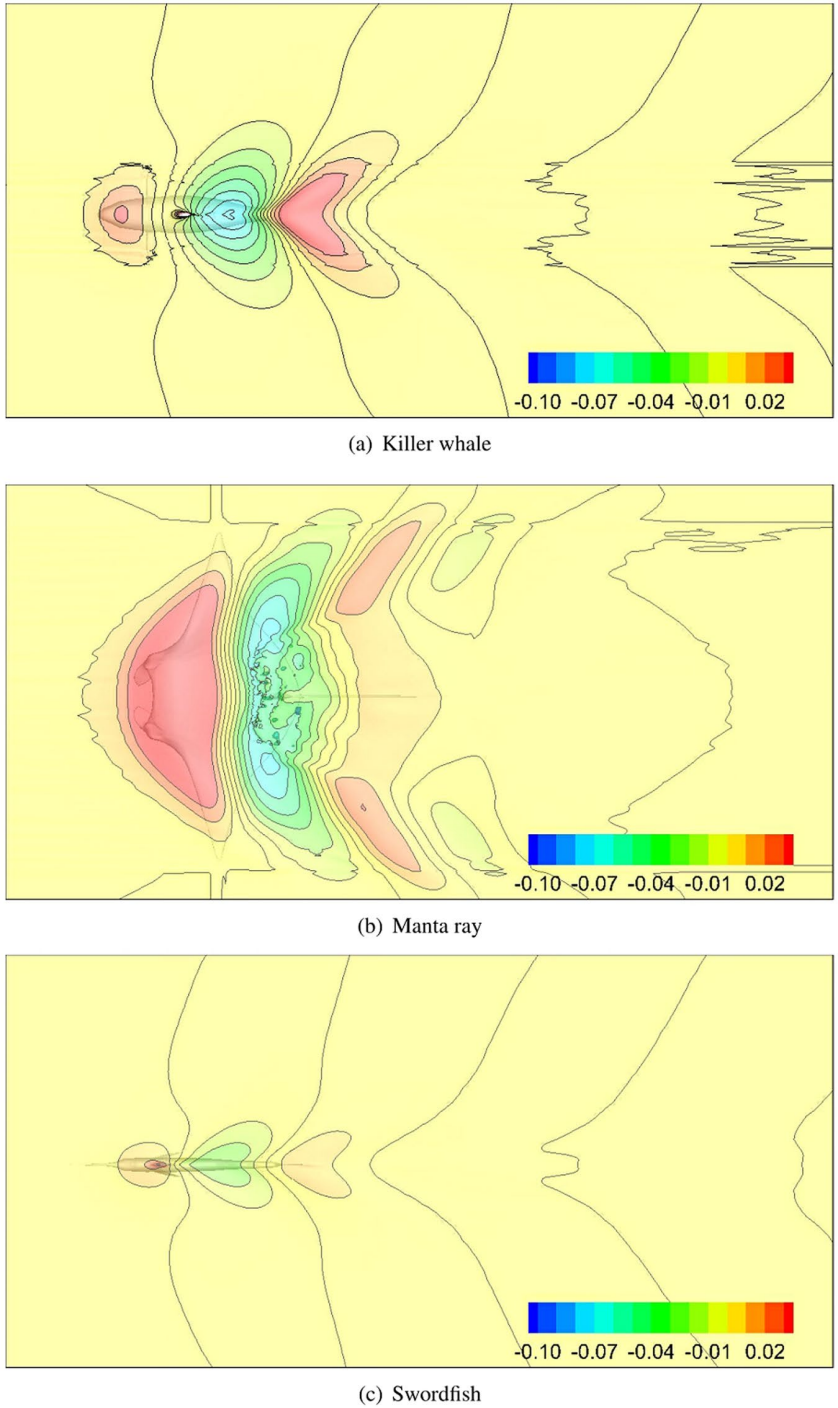
Top view of water-air surface for Case NS. Colored section corresponds to (*Z* − *Z*_0_)/*L*, where Z is the z-coordinate, and *Z*_0_ is the initial surface elevation. The CAD models are created in Rhinoceros^17^.

**Figure 6:**
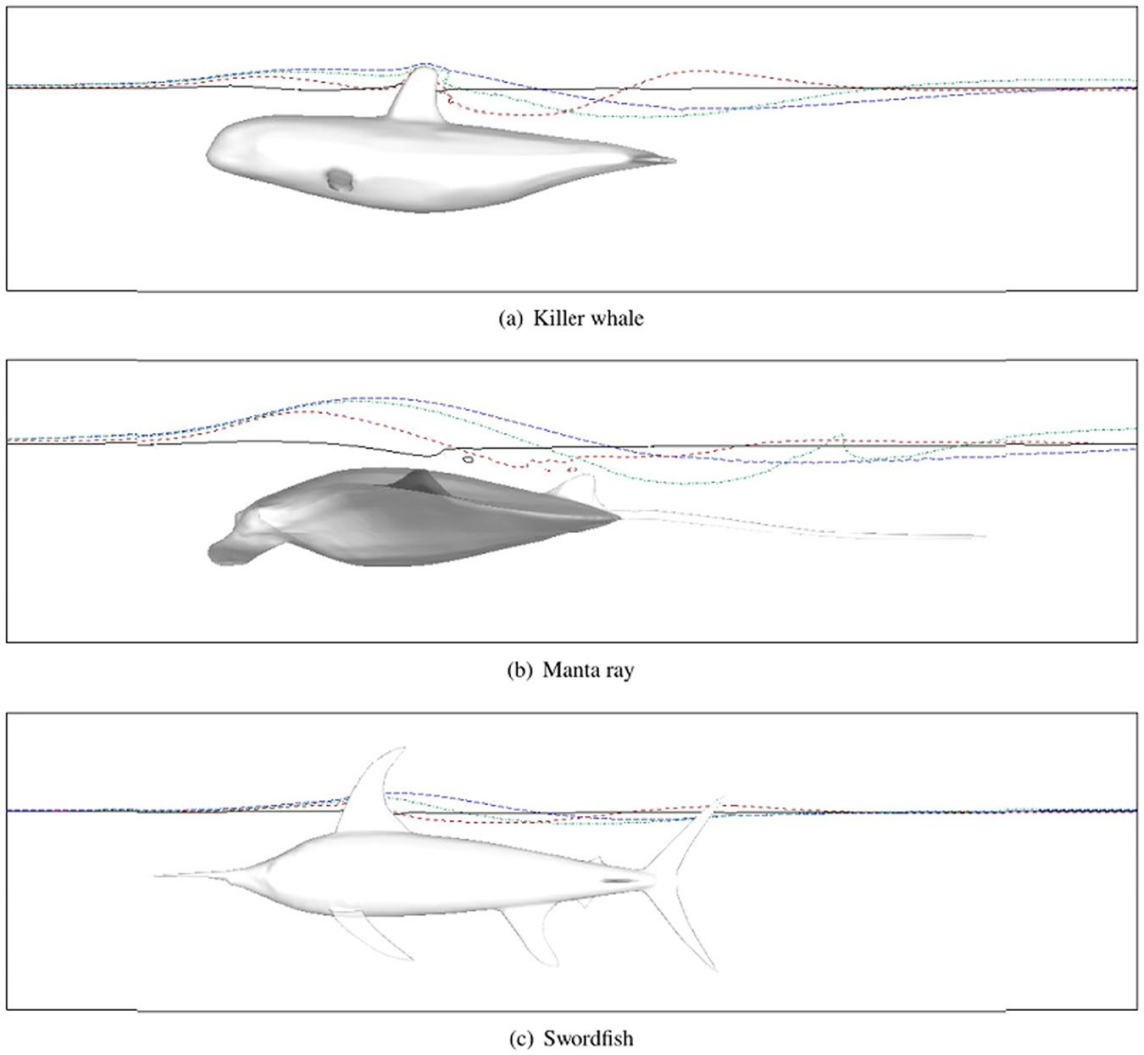
Instantaneous wave profile for various Froude numbers for Case NS at the beginning of the wave. The four lines are ordered from bottom to top with increasing swimming speed U = 0.5, 1.5, 2.5, and 3.5 m/s. The CAD models are created in Rhinoceros^17^.

Figure 5 shows the wave profiles for the three marine animal models at *U* = 1.5 *m*/*s*. Among the three scaled models, the wave fluctuation on the free surface is the smallest for the best streamlined swordfish with the smallest wave drag coefficient. The induced wave amplitudes of the three target models follow the same order of their wave drag coefficients. This is confirmed by Fig. 6, which shows the free surface lines at various swimming speeds. Here, the free surface line corresponds to the line of intersection of the iso-surface (volume fraction = 0.5) with the X-Z plane. For all three target models, the free surface lines on the left side of the turning points are ordered from bottom to top by increasing swimming speed U = 0.5, 1.5, 2.5, and 3.5 m/s.

Additionally, we observed in Fig. 5 that the wave profile of the manta ray is different to killer whale and swordfish. Wave profile of the swordfish and the killer whale is very similar: a wave crest occurs above the head region of the body; a heart-like wave valley occurs above the rear section; and another heart-like wave crest occurs in the wake of the tail. However, the heart shape of the wave valley above the rear section of the manta ray is broken, and even leading to the attenuation of its wake, as shown in Fig. 5. Figure 6 shows that the free surface lines above the killer whale and the swordfish are disturbed by their surface-piercing dorsal fin. For manta ray, the dorsal fin is very small, but wave breaking is still observed upon the rear section at certain swimming speeds (U < 2.0 m/s), when the wave drags are relatively larger.

**Figure 7:**
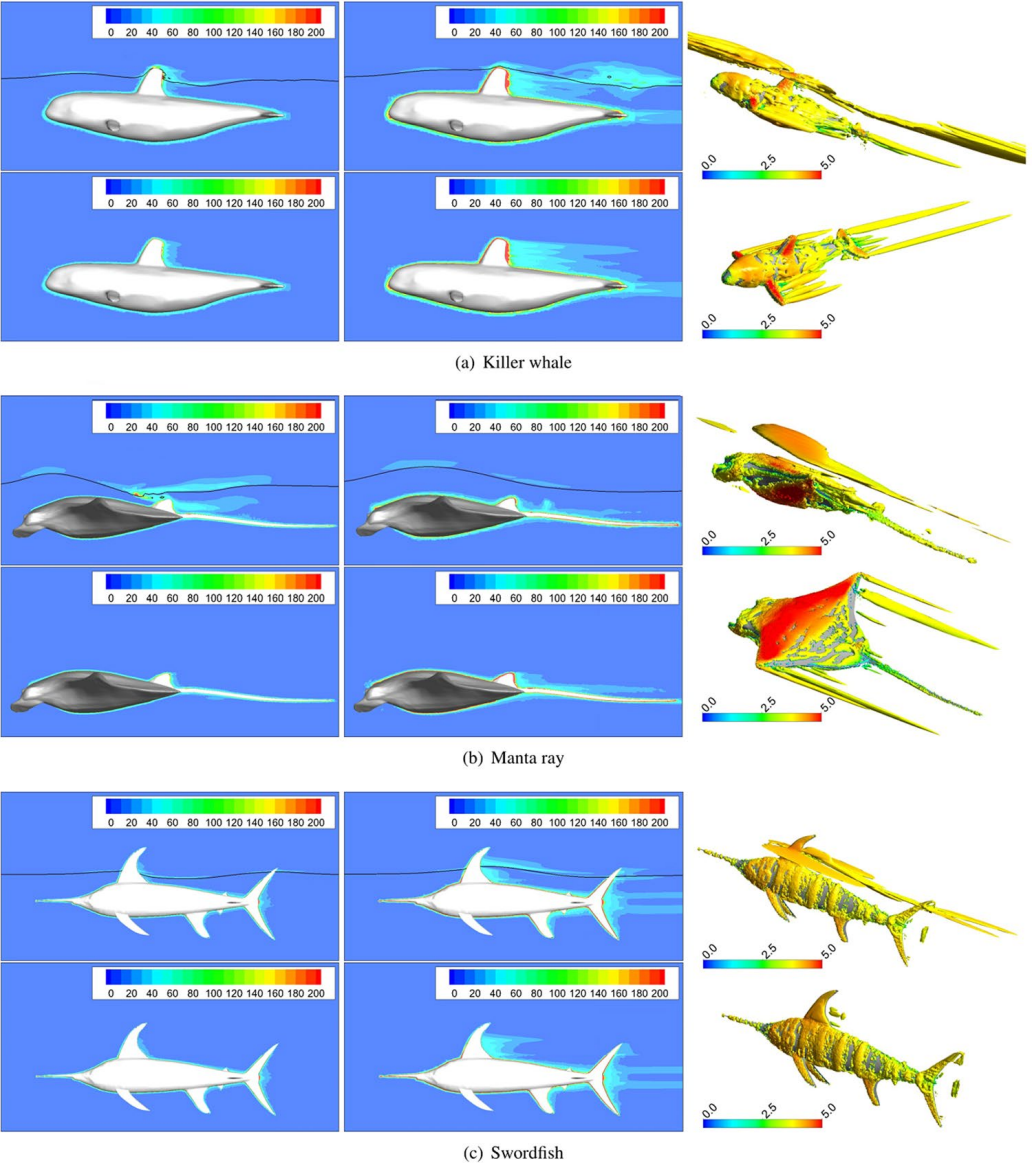
Cross-sectional plot for three target models. Top row: Case UW; bottom row: Case NS, where black lines correspond to the free surface, i.e. the intersection between the iso-surface (volume fraction = 0.5) and cross-sectional plane; 1st column: magnitude of the vorticity at U = 1.5 m/s; 2nd column: magnitude of the vorticity at U = 4.0 m/s; 3rd column: iso-surface of the swirling strength *W* = 0.01 *s* ^−1^ at U = 4.0 m/s, and the iso-surface is colored by the magnitude of the velocity. The CAD models are created in Rhinoceros^17^.

A more in-depth comparison was conducted using the cross-sectional plots of the magnitude of vorticity at U = 1.5 and 4.0 m/s and the iso-surface of the the swirling strength *W* = 0.01 *s* ^−1^ at U = 4.0 m/s, as shown in Fig. 7. For the killer whale, abundant vortex is observed behind the dorsal fin, pectoral fin, tail fin and the disturbed free surface above the body. For the swordfish with the smallest wave drag coefficient, though the vortex size is smaller, one vortex ring is observed behind the tail fin, and one fish tail shaped vortex in the wake region on the free surface. For the manta ray with a long tail, vortex is mainly distributed around the front section of the body. Its special body shape limits the production of the wake wave as killer whale and manta ray.

## Discussion

Three large size and fast speed marine animals, killer whale, manta ray and swordfish, are selected for this study for their different body shape. The characteristic lengths of the scaled models are unified as 1 m to eliminate the size effect. The hydrodynamics of the three target models in free surface flow are resolved using the VOF method and the RNG *k* − *ε* turbulence model. To validation the numerical results, one physical model of the manta ray is built using 3D printing technology, and its drag coefficient is measured in a towing tank. The numerical simulated drag coefficients of the physical model with towing speed U = 0.5, 1.0, $\cdots$, and 3.0 m/s are in good accordance with the experimental measurements.

Either in Case NearSurface or Case UnderWater, the numerical calculated drag coefficients of the three marine animal models are always ordered as, manta ray > killer whale > swordfish. In nature, the swimming speeds of the three target marine animals are also exactly in the reverse order, manta ray < killer whale < swordfish. Note that after excluding the size effect, the calculated friction drag coefficients of the three target models are very similar. Hence, the difference in passive drag is mainly accounted for by the difference in pressure drag, which is approximated by the sum of the form drag and the wave drag. In Case NearSurface, the wave drag is nearly zero, and the body shape is the main factor behind the variation of the passive drag and the affected natural swimming speed. In terms of swimming speed, the most streamlined body shape of the swordfish is the most competitive.

In Case NearSurface, the wave drag of the swordfish is also the smallest. However, its surface-piercing dorsal fin also cuts through the water surface and induces wave breaking as the killer whale. The wake wave introduced by their gliding locomotion can be transported long distance. For the flat and diamond-shaped manta ray with two spread-out and delta-shaped wings, the wave drag is extremely large at U = 0.5, 1.0 and 1.5 m/s, although the dorsal fin of the manta ray does not pierce the water surface as killer whale and swordfish. One noteworthy point is that the transporting of the wake wave behind the manta ray is broken by the wave breaking upon its rear section. Gliding locomotion of the manta ray near the free surface does not produce the wake wave as killer whale and swordfish. This is possibly the reason that why the manta ray can access fishing boats undetected and surprise the fishermen.

## Materials and Methods

### Target model

Three virtual marine animal models are generated using a Computer-aided Industrial Design (CAID) software, Rhinoceros^17^, as shown in Fig. 1. The 3D models are built based on the 2D photos taken from different angles (the source of photograph is several open source website, e.g. http://fishbase.org/). The target model is not exactly same to one real marine animal. Minor modification is made for the convenience of computation and experiment. For example, the target model is set up to be axisymmetric, while a real fish-like marine animal is always asymmetric.

**Figure 8:**
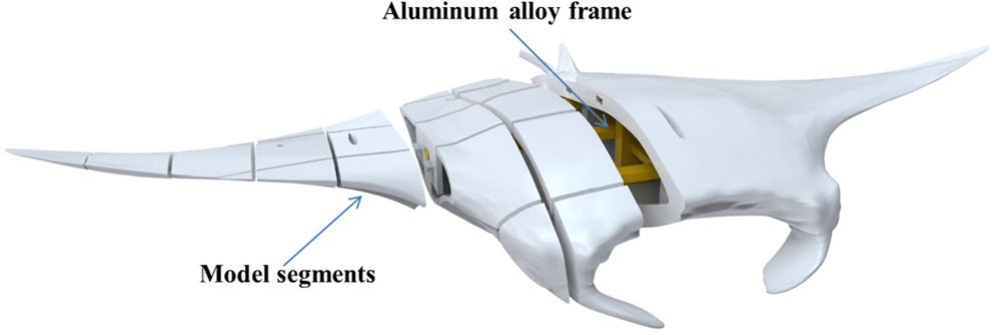
Schematic diagram of the 3D manta ray model.

Then a full-scale solid model of the manta ray is built using several advanced 3D printers, Ultimaker 2 extended+, which can offer the largest print size of 223 mm * 223 mm * 305 mm, as shown in Fig. 8. Economical and environmentally friendly PLA (Polylactice Acid) material is used for 3D printing. The manta ray test model is divided into 50 segments, such that the size of the segment does not exceed the printer job size limitation. Each segment is bonded together through the two-component epoxy resin glue. In addition, one aluminum alloy frame with excellent corrosion resistance is used to reinforce the model.

### Experiment design

**Figure 9:**
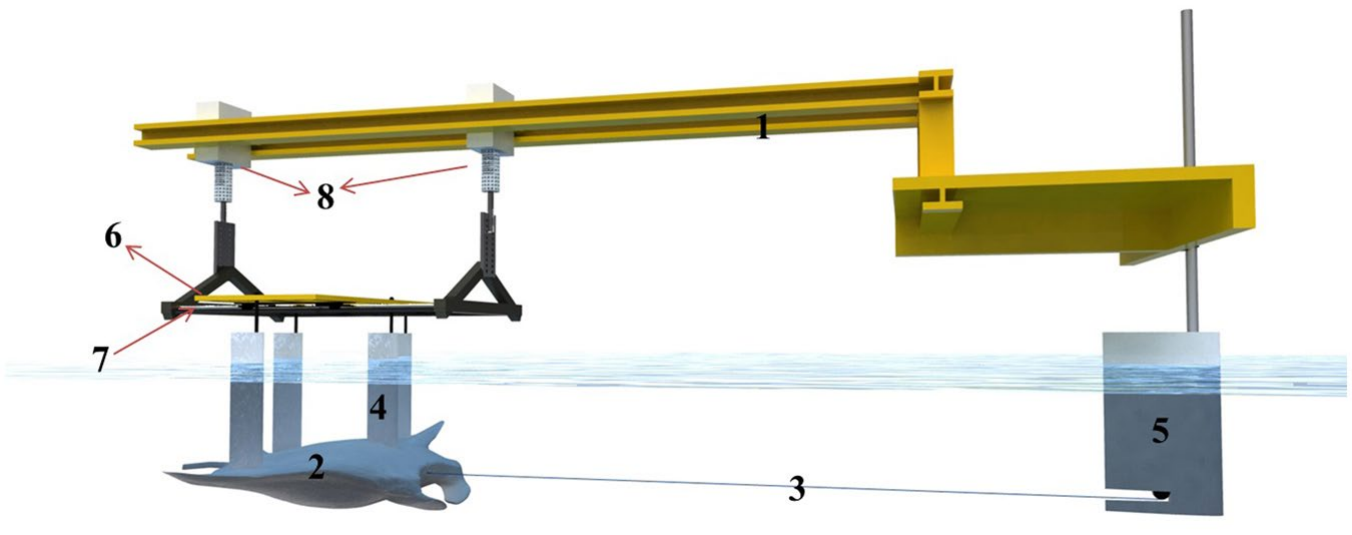
Schematic diagram of the towing test: 1. Carriage; 2. Manta ray model; 3. Rag force line; 4. Tear-shaped columns; 5. Front tear-shaped column; 6. Longitudinal motion support; 7. Longitudinal guide rod; 8. Elevator.

The manta ray resistance test is carried out in a towing tank with a length of 206 m, a width of 6 m and a water depth of 2.5 m at Sun Yat-sen University, as shown in Fig. 9. The test model is suspended from a movable support by four tear-shaped columns, and the support can be moved in one degree of freedom along the longitudinal guide rod. The movable support is connected to a carriage providing by two adjustable height elevators located at both ends. The towing speed varies in the range of 0 *m*/*s* < *U* ≤ 3 *m*/*s*, limited by the tank length. The manta ray model is placed at a distance of 0.24 m from the hydrostatic surface. The resistance of the manta ray is measured by one horizontal force line hanging at the front of the model. The other end of the force line is connected to a tear-shaped column, and the horizontal distance between the tear-shaped column to the front of the model is 2.4 m.

### Mathematical modeling

Under the RANS framework, the governing Navier-Stokes equations for the incompressible and viscous fluid flow are time averaged as below:

5 $$\frac{\partial \rho }{\partial t}+\nabla \cdot (\rho {\bf{u}})=0,$$

6 $$\frac{\partial \rho {\bf{u}}}{\partial t}+\nabla \cdot (\rho {\bf{uu}})=-\nabla \cdot p+\nabla \cdot ({\tau }_{ij}+{\tau }_{ij}^{R})+\rho {\bf{g}},$$

where *ρ*, **u** and *τ*_*ij*_ denotes the time averaged fluid density, velocity and stress tensor, respectively. On the right side of Eq. (6), *p* represents the pressure and **g** denotes the gravitational acceleration. According to the Boussinesq assumption, the Reynolds-stress *τ* ^*R*^ is assumed to linearly relate to the the mean flow straining field as following,

7 $${\tau }^{R}\equiv -\rho \tilde{{u}_{i}^{^{\prime} }{u}_{j}^{^{\prime} }}=2{\mu }_{t}{S}_{ij}-\frac{2}{3}\rho k{\delta }_{ij},$$

where **u**′ is the fluctuation part of the velocity, $k\equiv \frac{1}{2}\tilde{{u}_{j}^{^{\prime} }{u}_{j}^{^{\prime} }}$ is the mean turbulent kinetic energy, and $\tilde{\cdot }$ represents the Favre time averaging^26^. The turbulent viscosity *μ*_*t*_ is modeled as *μ*_*t*_ = *C*_*μ*_ *ρk* ^2^/*ε* with a default constant *C*_*μ*_ = 0.0845. The mean strain rate is defined by

8 $${S}_{ij}\equiv \frac{1}{2}(\frac{\partial {u}_{i}}{{x}_{j}}+\frac{\partial {u}_{j}}{{x}_{i}})-\frac{1}{3}\frac{\partial {u}_{k}}{{x}_{k}}{\delta }_{ij}.$$

The two new turbulent variables, turbulent kinetic energy *k* and dissipation rate *ε*, are resolved by the RNG (renormalization-group) *k* − *ε* closure equations^27^ as below.

9 $$\begin{array}{rcl}\frac{\partial \rho k}{\partial t}+\nabla \cdot (\rho {\bf{u}}k) & = & \nabla \cdot {\mu }_{\alpha }\nabla k+\mu {S}^{2}-\rho \varepsilon \\ \frac{\partial \rho \varepsilon }{\partial t}+\nabla \cdot (\rho {\bf{u}}\varepsilon ) & = & \nabla \cdot {\mu }_{\alpha }\nabla \varepsilon -(\frac{2}{3}{C}_{\varepsilon 1}-{C}_{\varepsilon 3})\rho \varepsilon \nabla \cdot {\bf{u}}+\frac{\varepsilon }{k}[{C}_{\varepsilon 1}^{\ast }{\mu }_{t}{S}^{2}-{C}_{\varepsilon 2}\rho \varepsilon ],\end{array}$$

where $S=\sqrt{2{S}_{ij}{S}_{ij}}$ represents the modulus of the mean strain rate tensor, *μ*_*α*_ = *α*_*k*_ *μ*_*t*_ + *μ* with *α*_*k*_ = 1.39, *C*_*ε*2_ = 1.92, *C*_*ε*3_ = −1.0, ${C}_{\varepsilon 1}^{\ast }={C}_{\varepsilon 1}-{C}_{\eta }$ with *C*_*ε*1_ = 1.44 and *C*_*η*_ = *η*(1 − *η*/*η*_0_)/(1 + *βη* ^3^) with *η*_*0*_ = 4.38, *β* = 0.012 and *η* = *Sk*/*ε*. The governing Navier-Stokes equations were solved numerically using a commercial CFD modeling package, ANSYS FLUENT 15^28^, with PISO (Pressure implicit with splitting of operator) as the pressure-velocity coupling algorithm.

To track the deformation of the air-water interface, the VOF method introduced one new transport equation for the volume fraction of liquid *α*_1_:

10 $$\frac{\partial {\alpha }_{1}}{\partial t}+\nabla \cdot ({\alpha }_{1}{\bf{u}})=0.$$

The volume fraction of air, *α*_2_, satisfies *α*_1_ + *α*_2_ ≡ 1. Then any material property (e.g., density and viscosity), *ϕ*, of the mixture can be evaluated as following,

11 $$\varphi ={\varphi }_{1}{\alpha }_{1}+{\varphi }_{2}{\alpha }_{2}.$$

### Boundary conditions

The boundary conditions are designed to represent two-phase flow in a channel with its top open to the air. The inlet of the open channel flow is specified to be a uniform velocity distribution with the value *U* shown in Table 1. Specific turbulent values are carefully applied at the inlet to ensure that the boundary values are physical and do not impede convergence. The turbulent intensity *I* of the open channel flow is given by

12 $$I=0.16{(R{e}_{{D}_{H}})}^{-\mathrm{1/8}}.$$

where *L* is the body length, *ρ* is the fluid density, and *μ* is the fluid viscosity. The Reynolds number $R{e}_{{D}_{H}}$ is given by

13 $$R{e}_{{D}_{H}}=\frac{\rho U{D}_{H}}{\mu },$$

where the hydrodynamic diameter *D*_*H*_ is defined as,

14 $${D}_{H}=\frac{2{d}_{W}{W}_{D}}{2{d}_{W}+{W}_{D}}.$$

The computation domain width *W*_*D*_ and water depth *d*_*W*_ are given in Table 1. Then, the inlet turbulent kinetic energy *k* and the dissipation rate *ε* are calculated by

15 $$k=\frac{3}{2}{(UI)}^{2}{\rm{and}}\,\varepsilon ={C}_{\mu }^{\mathrm{3/4}}\frac{{k}^{\mathrm{3/2}}}{{D}_{H}},$$

with the constant *C*_*μ*_ = 0.09.

The pressure at the outlet has a zero gradient. The two sides and bottom of the computational domain are slip walls with zero shear, i.e. the wall has negligible effect on the fluid motion. A pressure outlet boundary condition at atmospheric pressure is imposed at the top of the domain. No-slip wall conditions are applied at the surface of the body.

### Computation setup

**Figure 10:**
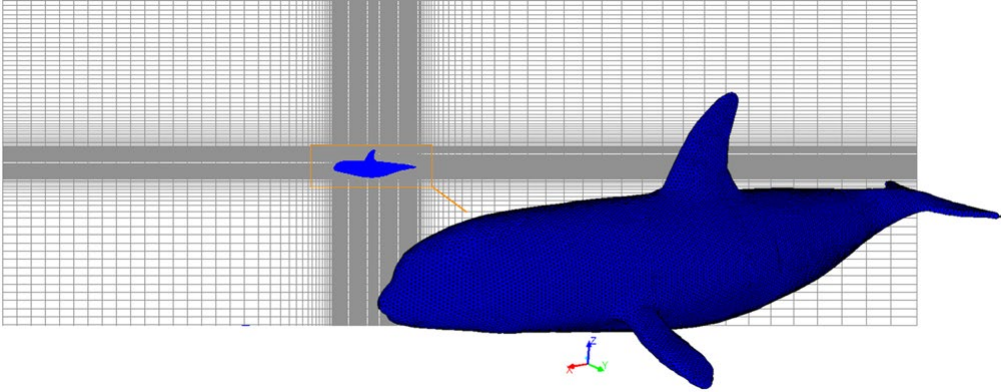
Computational mesh showing a detailed surface mesh for the killer whale.

Then Rhinoceros exports files with a three-dimensional STEP format (*.stp) into Gambit, which is a geometry and mesh generation software from Fluent. The complex geometry of the body requires the generation of unstructured tetrahedral cells around the body using Tgrid, which is an specialized ANSYS preprocessor. Hexahedron cells are used in the remaining computational domain, as shown in Fig. 10. The computation mesh is refined until the drag coefficient converges, and the relative numbers of computation cells are given in Table 1.

Table 1 lists the geometrical parameters, water depths and computational domain settings for the three target models. The wetted area *A* is the area of the body in contact with the fluid. The characteristic length *L*_0_ of each model is measured from the center of the eye to the starting point of the caudal fin. This measurement excludes the length of the snout and the caudal fin, considered the extreme long snout of the swordfish and the caudal fin of the manta ray. Here, the size of the model is adjusted such that *L*_0_ = 1 for each target h. In Case NearSurface, the dorsal fins of the killer whale and the swordfish are above the water surface, such that *A* ^*NS*^ of the killer whale is smaller than *A* ^*UW*^, as shown in Table 1. The length (L) of the model refers to the full length, which is measured from the tip of the snout to the end of the caudal fin. The body position *d* is taken to be the distance between the air-water interface and the center line of the model. Each target model is tested under various swimming speed $U=0.5\,m/s,1.0\,m/s,\cdots ,4.0\,m/s$.
